# Dying in long-term care facilities in Europe: the PACE epidemiological study of deceased residents in six countries

**DOI:** 10.1186/s12889-019-7532-4

**Published:** 2019-08-30

**Authors:** Elisabeth Honinx, Nanja van Dop, Tinne Smets, Luc Deliens, Nele Van Den Noortgate, Katherine Froggatt, Giovanni Gambassi, Marika Kylänen, Bregje Onwuteaka-Philipsen, Katarzyna Szczerbińska, Lieve Van den Block, on behalf of PACE, Yuliana Gatsolaeva, Rose Miranda, Lara Pivodic, Marc Tanghe, Hein van Hout, Roeline H. R. W. Pasman, Mariska Oosterveld-Vlug, Ruth Piers, Anne B. Wichmann, Yvonne Engels, Myrra Vernooij-Dassen, Jo Hockley, Sheila Payne, Suvi Leppäaho, Ilona Barańska, Sophie Pautex, Catherine Bassal, Federica Mammarella, Martina Mercuri, Paola Rossi, Ivan Segat, Agata Stodolska, Eddy Adang, Paula Andreasen, Outi Kuitunen-Kaija, Danni Collingridge Moore, Agnieszka Pac, Violetta Kijowska, Maud ten Koppel, Jenny T. van der Steen, Emilie Morgan de Paula

**Affiliations:** 1 Department of Family Medicine & Chronic Care, Vrije Universiteit Brussel (VUB) & Ghent University, Laarbeeklaan 103, 1090 Brussels, Belgium; 2 Department of Geriatric Medicine, Ghent University Hospital, De Pintelaan 185, 9000 Ghent, Belgium; 3 International Observatory on End of Life Care, Faculty of Health and Medicine, Lancaster University, Lancaster, LA1 4YW UK; 4 Department of Internal Medicine, Istituto di Medicina Interna e Geriatria, Università Cattolica del Sacro Cuore, Largo F. Vito, 1, 00135 Rome, Italy; 5 National Institute for Health and Welfare, Mannerheimintie 166, P.O. Box 30, FI-00271 Helsinki, Finland; 6 Department of Public and Occupational Health, EMGO Institute for Health and Care Research, Expertise Center for Palliative Care, VU University Medical Center, Van der Boechorstraat 7, 1081 BT Amsterdam, The Netherlands; 7 Department of Sociology of Medicine, Chair of Epidemiology and Preventive Medicine, Medical Faculty, Jagiellonian University Medical College, ul. Kopernika 7a, 31-034 Kraków, Poland

**Keywords:** End-of-life care, Long-term care facility, Nursing homes, Palliative care, Policy

## Abstract

**Background:**

By 2030, 30% of the European population will be aged 60 or over and those aged 80 and above will be the fastest growing cohort. An increasing number of people will die at an advanced age with multiple chronic diseases. In Europe at present, between 12 and 38% of the oldest people die in a long-term care facility. The lack of nationally representative empirical data, either demographic or clinical, about people who die in long-term care facilities makes appropriate policy responses more difficult. Additionally, there is a lack of comparable cross-country data; the opportunity to compare and contrast data internationally would allow for a better understanding of both common issues and country-specific challenges and could help generate hypotheses about different options regarding policy, health care organization and provision. The objectives of this study are to describe the demographic, facility stay and clinical characteristics of residents dying in long-term care facilities and the differences between countries.

**Methods:**

Epidemiological study (2015) in a proportionally stratified random sample of 322 facilities in Belgium, Finland, Italy, the Netherlands, Poland and England. The final sample included 1384 deceased residents. The sampled facilities received a letter introducing the project and asking for voluntary participation. Facility manager, nursing staff member and treating physician completed structured questionnaires for all deaths in the preceding 3 months.

**Results:**

Of 1384 residents the average age at death ranged from 81 (Poland) to 87 (Belgium, England) (*p* < 0.001) and length of stay from 6 months (Poland, Italy) to 2 years (Belgium) (*p* < 0.05); 47% (the Netherlands) to 74% (Italy) had more than two morbidities and 60% (England) to 83% (Finland) dementia, with a significant difference between countries (p < 0.001). Italy and Poland had the highest percentages with poor functional and cognitive status 1 month before death (BANS-S score of 21.8 and 21.9 respectively). Clinical complications occurred often during the final month (51.9% England, 66.4% Finland and Poland).

**Conclusions:**

The population dying in long-term care facilities is complex, displaying multiple diseases with cognitive and functional impairment and high levels of dementia. We recommend future policy should include integration of high-quality palliative and dementia care.

## Background

By 2030, 30% of the European population will be aged 60 or over and those aged 80 and above will be the fastest growing cohort [1]. An increasing number of people will die at an advanced age with multiple chronic diseases [2–4]. In Europe at present, between 12 and 38% of the oldest people die in a long-term care facility [5]. The term long-term care facility in this study is used for all ‘collective institutional settings where care, on-site provision of personal assistance with activities of daily living, and on-site or off-site provision of nursing and medical care, is provided for older people who live there 24 hours a day, 7 days a week, for an undefined period of time’^6^ .There are many types of long-term care facilities, due to the different health care systems and funding mechanisms in different countries [6]. Also, the number of older people in need of high quality end-of-life care in such facilities is increasing [2, 4]. Studies of the complexity of challenges posed by people spending their end-of-life period in long-term care facilities have so far been small-scale and limited to specific regions or illnesses [5–8].

The lack of nationally representative empirical data, either demographic or clinical, about people who die in long-term care facilities makes appropriate policy responses more difficult [7]. There are very few statistics comparable across Europe on the prevalence of dementia and multimorbidity, on functional and cognitive status or on clinical complications at the end of life in the residential setting. This lack of data makes it difficult for policy and decision-makers to gain insight into the key challenges of this population and provides them with few opportunities to monitor changes over time. Additionally, there is a lack of comparable cross-country data; the opportunity to compare and contrast data internationally would allow for a better understanding of both common issues and country-specific challenges and could help generate hypotheses about different options regarding policy, health care organization and provision.

Palliative Care for Older People (PACE) is an EU-funded project (2014–2019) which set out to conduct comparative research on older people dying in long-term care facilities in Europe. It is the first study aiming to describe and compare the characteristics of dying residents across 6 European countries, Belgium, the Netherlands, England, Finland, Italy and Poland. These countries were selected to reflect a variety of health care systems and geographic regions in Europe [9, 10].

The main research questions are [1]: what are the demographic and facility-stay characteristics of people who die in long-term care facilities and how do they differ between the 6 participating countries, and [2] what are the clinical characteristics of the residents who die there, including the prevalence of dementia and other conditions, their functional and cognitive status 1 month before death and the clinical complications during the last month of life, and how do these differ between the 6 countries.

## Methods

### Study design

An epidemiological study of deceased residents in long-term care facilities was conducted in Belgium, the Netherlands, England, Finland, Italy and Poland in 2015 [9]. To obtain representative samples of facilities, a proportional stratified random sampling procedure was used within each country. Based on available national or regional lists of all long-term care facilities, facilities were randomly and proportionally selected from several strata (based on at least region/province and facility size by beds). In Belgium and the UK, a sample was drawn from the region where most of the population lives (Flanders and England respectively). In England, the nationwide ENRICH (Enabling Research in Care Homes) network was used to increase participation of facilities. In Italy there was no public national list available and instead a convenience sample was used based on a previously constructed cluster of facilities interested in research, which covered the 3 macro regional areas and took into account different sizes and types of facilities.

In each country, participating facilities reported every death that occurred among the residents of their facility over the preceding 3-month period. Deceased residents were included in the study when death occurred in the facility, as were those whose deaths were registered outside (e.g. in acute care hospitals).

More details about the study design and protocol have been published [9].

### Setting and participants

Several types of long-term care facilities can be distinguished within the 6 countries, depending on whether care by specific health care professionals is provided on-site or off-site (see Table 1) [6, 9]. Type 1 includes facilities with 24 h care from on-site physicians, nurses and care assistants, type 2 are facilities with 24 h care from on-site nurses and care assistants and care from physicians who are based off-site and type 3 consists of facilities with 24 h on-site care from care assistants and care from nurses and physicians who are based off-site.

**Table 1:** Available types of facilities in six countries

	Type 1 facilities with on-site physicians, nurses and care assistants	Type 2 facilities with on-site nurses and care assistants and off-site physicians	Type 3 facilities with on-site care assistants and off-site nurses and physicians
Belgium		X	
The Netherlands	X	X	
The United Kingdom		X	X
Finland		X	
Italy	X	X	
Poland	X	X	

For each identified deceased resident, structured questionnaires were sent to the facility administrator/manager, the nursing staff member most involved in care (preferably a nurse) and the treating physician (TP; a general practitioner, elderly care physician or physician employed in the facility). For each participating facility, the administrator/manager was also asked to fill out a questionnaire on facility characteristics.

### Data collection

The long-term care facilities that were sampled in each country received a letter introducing the PACE project and asking for voluntary participation. Additional contact was made by phone or e-mail. Each participating facility appointed a contact person and was visited by a researcher. During this visit, the contact person listed all residents who had died in the preceding 3 months and identified 3 key respondents for each deceased resident (facility administrator/manager, nursing staff member, treating physician and relative) with the use of a structured checklist. Questionnaires were sent to the key respondents and up to 2 reminders were sent to non-responders (after 3 and 6 weeks). To ensure high-quality data collection, researchers in all countries were trained to follow a quality assurance manual designed for this project.

### Measurements

After-death questionnaires included validated instruments and were forward-backward translated according to European Organisation for Research and Treatment of Cancer guidelines [11] in cases where official translations did not exist.

The questionnaire for the facility administrator/manager included questions about the resident’s age, sex, residency before admission, length of stay, placement in ward or unit for residents with dementia at the time of death, place and cause of death, ownership (public, private-nonprofit or private-profit) and size of the facility.

The questionnaire for the nursing staff member included questions about the presence of dementia at the time death in the staff member’s opinion, the clinical complications during the last month of life (pneumonia, febrile episode, eating or drinking problem, hip fracture, stroke, gastrointestinal bleeding) and the functional and cognitive status of the resident 1 month before death. The Bedford Alzheimer Nursing Severity Scale (BANS-S) [12], the Global Deterioration Scale (GDS) stage 7 yes/no [13] and the Cognitive Performance Scale (CPS) [14]were used. The GDS classifies dementia into 7 stages based on cognition and function: Stage 7 is described as very severe cognitive decline with minimal to no verbal communication, assistance needed with toileting and feeding, incontinence and loss of basic psychomotor skills. The CPS assigns residents to cognitive performance categories, ranging from borderline intact to very severe impairment.

The questionnaire for the treating physician included questions on the presence of dementia and other diseases at the time of death in the physician’s opinion (malignant cancer, severe cardiovascular disease, cerebrovascular accident, severe pulmonary disease, severe neurological disease, severe renal disease, severe diabetes, other severe disease) and the presence of multimorbidity (counting the number of diseases at time of death from the 8 diseases listed above).

### Statistical analysis

All analyses were performed with SPSS 23. Preliminary analyses included creating a variable for dementia that does not underestimate its prevalence. When either the physician or the nurse (or both) considered the resident to have dementia, this was coded as ‘yes’. Stage of dementia was based on the CPS and GDS scores, as answered by nursing staff, after selecting residents with dementia: CPS ≥ 5 and GDS =7 was classified as ‘very severe or advanced dementia’, CPS ≥ 5 and GDS < 7 or CPS ≤ 5 and GDS = 7 as ‘severe dementia’, CPS ≤ 5 and GDS < 7 as ‘moderate or mild dementia’ [15].

All primary analyses had to account for the clustering of the data (in countries, facilities and physicians or nurses), thus a multilevel model was created for each analysis. Depending on the outcome or target variable (continuous, binary or categorical), generalized linear mixed models were designed with a normal, binomial or multinomial distribution. Country was included as a fixed effect in each model in order to test for differences between the 6 countries. The alpha level of α = 0.05 defines statistical significance. Facility was included as a random effect in each model. For variables that were based on questions answered by physicians or nursing staff, either physician or nursing staff member was added as a random effect. Significance of random effects are not reported as this is not the main focus of this paper.

Because countries, except for Belgium and Finland, had different facility types, we additionally conducted multilevel models per country for each analysis with facility type used as a fixed effect.

### Ethical aspects

The study protocol was approved by the relevant ethics committee in 2015 in each country or waivers for the collection of data of deceased residents were obtained (the Netherlands and Italy). Participation was completely anonymous and voluntary.

## Results

**Fig. 1:**
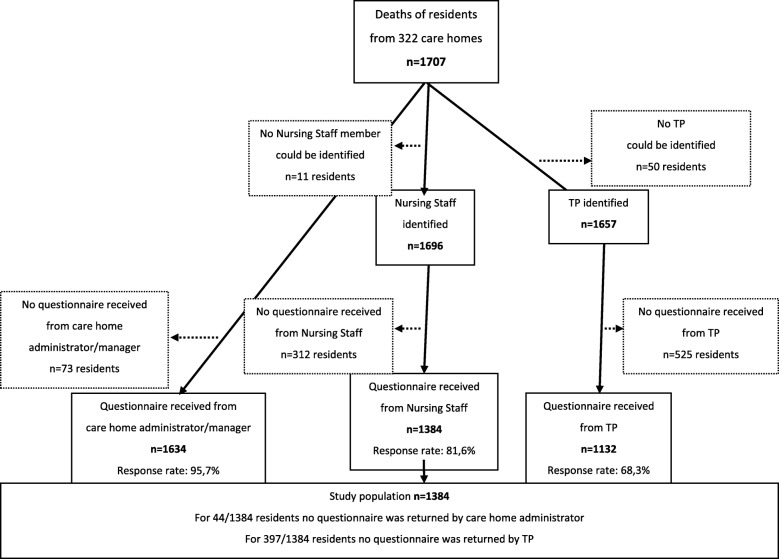
Numbers of questionnaires (distributed and returned) regarding residents per respondent in 6 countries

In total in the 6 countries, 322 long-term care facilities participated. Participating facilities identified 1707 residents who had died within the previous 3 months. Average response rates to the questionnaires for facility manager/administrators were 95.7% (Belgium 94.2%, the Netherlands 90.6%, England 96.4%, Finland 98.6%, Italy 96.5%, Poland 98.9%), for nursing staff 81.6% (Belgium 85.1%, the Netherlands 67.5%, England 54.2%, Finland 95.1%, Italy 91.7%, Poland 87.4%) and for treating physicians 68.3% (Belgium 66.9%, the Netherlands 63.1%, England 23.8%, Finland 80.2%, Italy 88.4%, Poland 75.6%). Figure 1 provides an overview of the total numbers of questionnaires in 6 countries that were distributed and returned per respondent. The final sample of interest accounting for all missing information was 1384 deceased residents.

### Non-response analysis

Using data provided by the facility administration, non-response analysis showed no differences for important demographic characteristics of the residents (age, sex, length of stay, place of death) between participating and non-participating nursing staff (data not shown). Between participating and non-participating physicians, non-response analysis showed similar results except for place of death (*p* = 0.04). The physicians more often participated in cases where the resident died in the facility than when they died outside the facility.

### Demographic and facility stay characteristics of dying facility residents

The mean age of the residents at the time of death was over 85 years except for those in Poland where it was 81 (Table 2). About 2/3 of the residents were female (63.5 to 75.0%) with no significant differences between countries. Most residents studied lived in a facility with on-site care from nurses and care assistants but off-site care from physicians. In the Netherlands and Poland however, most lived in a facility with on-site care from physicians, nurses and care assistants (*p* < 0.001). The facilities in which they lived also differed in size from an average of 41 beds in Finland and England to 126 beds in Belgium (*p* < 0.001). They also differed in type of ownership (*p* < 0.001); in most countries, the largest proportion of residents lived in public non-profit facilities, except for Italy and England where more stayed in private for-profit facilities (41.8 and 86.8% respectively). Most of the residents were admitted to the facility from their own home (30.0 to 57.9%) and a large proportion from a hospital (25.9 to 34.2%) except in the Netherlands and Italy where fewer residents were admitted from hospital (8.8% in the Netherlands and 16.8% in Italy; *p* < 0.001). The median length of stay in the facility ranged from less than 6 months in Poland to over 2 years in Belgium (*p* = 0.025). At the time of death, most residents were not living in a ward or unit specifically designed for residents with dementia. The largest proportion of residents who did stay in a dementia ward was found in the Netherlands (47.5%) and the smallest proportion in Poland (18.4%). Between 80% in of the residents in Poland and 90% of the residents in the Netherlands died in the facility and no significant differences were found between the countries regarding place of death.

**Table 2:** Demographic and Facility Stay Characteristics of Dying Facility Residents in Six European countries: (*N* = 1384)

	BE *N* = 291 *N*(%)	NL *N* = 222 *N*(%)	UK *N* = 91 *N*(%)	FI *N* = 269 *N*(%)	IT *N* = 200 *N*(%)	PL *N* = 311 *N*(%)	*P*-value*
Age - yr^a^							
Mean (SD) years old at time of death	87 (7)	86 (9)	87 (9)	85 (9)	86 (8)	81 (11)	**< 0.001**
Sex^a^							
Female	174 (64.0)	132 (66.7)	66 (75.0)	169 (64.3)	136 (68.3)	195 (63.5)	0.387
Male	98 (36.0)	66 (33.3)	22 (25.0)	94 (35.7)	63 (31.7)	112 (36.5)	
Type of ownership of facility^a^							
Public-nonprofit	135 (48.9)	211 (100.0)	2 (2.2)	211 (80.2)	66 (34.9)	201 (65.0)	**< 0.001**
Private-nonprofit	124 (44.9)	NA	10 (11.0)	24 (9.1)	44 (23.3)	104 (33.7)	
Private-profit	17 (6.2)	NA	79 (86.8)	28 (10.6)	79 (41.8)	4 (1.3)	
Size of facility^a^							
Mean (SD) number of beds	126 (50)	124 (64)	41 (23)	41 (30)	101 (50)	72 (41)	**< 0.001**
Residency before admission to facility^a^							
Own home (living alone or with family or others)	100 (41.8)	99 (57.9)	43 (50.6)	77 (30.0)	95 (49.7)	153 (49.8)	**< 0.001**
General hospital (e.g. acute care hospital)	76 (31.8)	15 (8.8)	22 (25.9)	85 (33.1)	32 (16.8)	105 (34.2)	
Other facility	32 (13.4)	32 (18.7)	18 (21.2)	72 (28.0)	32 (16.8)	35 (11.4)	
Other residency (e.g. psychiatric or rehabilitation hospital)	31 (13.0)	14.6)	2 (2.4)	23 (8.9)	32 (16.8)	14 (4.6)	
Length of stay^a^							
Median (min-max) number of days	745 (2–9706)	710 (1–6290)	600 (2–4952)	581 (1–9218)	416 (2–10,171)	145 (1–12,365)	**< 0.05**
Type of ward at time of death^a^							
Ward for residents with dementia	104 (38.1)	94 (47.5)	29 (33.3)	115 (43.7)	74 (37.9)	56 (18.4)	**< 0.01**
Ward not specifically for dementia	169 (61.9)	104 (52.5)	58 (66.7)	148 (56.3)	121 (62.1)	249 (81.6)	
Place of Death^a^							
Facility	226 (82.2)	176 (89.3)	71 (81.6)	224 (84.8)	170 (86.7)	248 (80.0)	0.922
Other (e.g. hospital)	49 (17.8)	21 (10.7)	16 (18.4)	40 (15.2)	26 (13.3)	62 (20.0)	

Missing values age = 13, sex = 13, type = 0, ownership = 6 size = 60, admission from = 145, length of stay = 36, type of ward dementia = 19, place of death = 11. Percentages may not always add up to 100 because of rounding

*Generalised linear mixed model reporting *p*-value for country as a fixed effect, **α** = 0.05. Significant results in bold

^a^Reported by administrator/manager of facility. For 44 out of 1384 residents no questionnaire was returned by the administrator/manager of facility; these are not included as missing values below.

### Clinical characteristics

In all 6 countries, more than 60% of the residents had dementia at the time of death according to their treating physician and/or nurse (Table 3). However, there was a significant difference between countries (*p* < 0.001) with the prevalence of dementia ranging from 60.2% in England to 82.5% in Finland. Among residents who died with dementia, no statistical difference between the countries was found for the stage of dementia 1 month before death. More than half of these had very severe or advanced dementia shortly before death in Poland (64.0%), Italy (55.0%) and Belgium (52.5%).

**Table 3:** Clinical Characteristics of Dying Facility Residents in Six European Countries (*N* = 1384)

	BE *N* = 291	NL *N* = 222	UK *N* = 91	FI *N* = 269	IT *N* = 200	PL *N* = 311	
	*N*(%)	*N*(%)	*N*(%)	*N*(%)	*N*(%)	*N*(%)	*P*-value*
Dementia at time of death (yes)^abc^	183 (62.9)	135 (61.4)	53 (60.2)	222 (82.5)	154 (77.0)	207 (67.9)	**< 0.001**
Stage of Dementia (based on CPS/GDS^d^) ^ab^							
Moderate or mild dementia	27 (17.1)	29 (22.3)	13 (31.0)	43 (24.2)	14 (11.7)	17 (11.3)	0.676
Severe dementia	48 (30.4)	41 (31.5)	11 (26.2)	57 (32.0)	40 (33.3)	37 (24.7)	
Very severe or advanced dementia	83 (52.5)	60 (46.2)	18 (42.9)	78 (43.8)	66 (55.0)	96 (64.0)	
Diseases at time of death^be^							
Malignant cancer^b^	30 (15.5)	27 (18.5)	9 (42.9)	41 (19.4)	26 (17.2)	10 (4.0)	**< 0.001**
Severe cardiovascular disease^b^	67 (34.7)	45 (30.8)	2 (9.5)	79 (37.4)	71 (47.0)	141 (55.7)	**< 0.05**
Cerebrovascular accident (CVA)^b^	40 (20.7)	25 (17.1)	3 (14.3)	49 (23.2)	34 (22.5)	70 (27.7)	0.483
Severe pulmonary disease^b^	33 (17.1)	17 (11.6)	3 (14.3)	17 (8.1)	40 (26.5)	18 (7.1)	**< 0.001**
Severe neurological disease (not dementia)^b^	15 (7.8)	11 (7.5)	0 (0.0)	26 (12.3)	18 (11.9)	32 (12.6)	0.381
Severe renal disease^b^	19 (9.8)	19 (13.0)	2 (9.5)	13 (6.2)	22 (14.6)	29 (11.5)	0.420
Severe diabetes^b^	11 (5.7)	17 (11.6)	1 (4.8)	16 (7.6)	18 (11.9)	33 (13.0)	0.177
Other severe disease^b^	31 (16.1)	4 (2.7)	3 (14.3)	51 (24.2)	33 (21.9)	33 (13.0)	**< 0.001**
Multimorbidities at time of death^b^							
0–1 long-term conditions	94 (48.7)	77 (52.7)	10 (47.6)	72 (34.1)	39 (25.8)	98 (38.7)	0.268
2 multimorbidities	54 (28.0)	40 (27.4)	8 (38.1)	68 (32.2)	52 (34.4)	102 (40.3)	
3 multimorbidities	30 (15.5)	25 (17.1)	2 (9.5)	49 (23.2)	37 (24.5)	40 (15.8)	
4 or more multimorbidities	15 (7.8)	4 (2.7)	1 (4.8)	22 (10.4)	23 (15.2)	13 (5.1)	
Functional/cognitive status one month before death (BANS-S)^af^							
Mean (SD)	18.5 (4.9)	17.7 (4.7)	17.5 (4.2)	19.6 (4.3)	21.8 (3.7)	21.9 (4.6)	**< 0.001**
Cognitive Performance Scale one month before death^ag^							
(Borderline) Intact (score 0–1)	75 (28.0)	42 (20.5)	17 (23.3)	23 (9.4)	16 (9.9)	72 (11.6)	**< 0.01**
Mild to moderate impairment (score 2–3)	55 (20.5)	58 (28.2)	21 (28.7)	56 (22.9)	27 (16.7)	37 (15.9)	
Moderately severe to very severe impairment (score 4–6)	138 (51.5)	105 (51.2)	35 (47.9)	165 (67.5)	118 (73.3)	169 (72.5)	
Global Deterioration Scale Stage 7 one month before death^ah^	155 (57.8)	104 (47.5)	40 (50.6)	111 (48.3)	124 (66.0)	208 (73.2)	**< 0.001**
Clinical complications during last month of life^a^							
Pneumonia^a^	64 (24.2)	75 (37.7)	16 (21.6)	76 (31.4)	45 (25.3)	62 (21.2)	**< 0.01**
Febrile episode (not pneumonia) ^a^	118 (44.2)	36 (18.4)	4 (5.4)	78 (31.1)	98 (52.4)	136 (46.6)	**< 0.001**
Eating or drinking problem^a^	179 (65.3)	125 (60.4)	41 (51.9)	170 (66.4)	113 (60.8)	198 (66.4)	0.237
Other clinical complications (e.g. stroke)^a^	96 (33.0)	81 (36.5)	38 (41.8)	65 (24.2)	81 (36.5)	113 (36.3)	**< 0.05**

Percentages may not always add up to 100 because of rounding

Missing values: dementia = 11, stage of dementia = 187 (419 not applicable because resident did not have dementia), diseases at time of death = 12, multimorbidities = 12, BANS-S = 86 missing data on at least one item, CPS = 182 (and 18 not applicable), GDS7 = 116, clinical complications = 81

Abbreviations: *CPS* Cognitive Performance Scale, *GDS* Global Deterioration Scale, *BANS-S* Bedford Alzheimer Nursing Severity Scale

*Generalised linear mixed model reporting *p*-value for country as a fixed effect, α =0.05. Significant results in bold

^a^Reported by staff member (nurse/care assistant) most involved in care

^b^Reported by treating physician (TP) For 397 out of 1384 residents no questionnaire was returned by the TP, these are not included as missing values below

^c^When either the physician or the nurse (or both) considered the resident to have dementia, this was coded as yes

^d^The variable stage of dementia was based on the scores on the CPS and GDS, as answered by nursing staff, after selecting residents with dementia [16]

^e^Multiple answers possible

^f^Scores on BANS-S range from 7 to 28; higher scores indicate greater severity

^g^Scores on CPS range from 0 to 6; higher scores indicate greater severity

^h^Scores on GDS range from 0 to 6; higher scores indicate greater severity

Other than dementia, the most prevalent disease at the time of death was severe cardiovascular disease in all countries (30.8–55.7% of residents) except England (9.5%) (*p* < 0.001), where nearly half of the residents had cancer (42.9%). For cerebrovascular accidents, severe neurological disease, severe renal disease and severe diabetes no significant differences between countries were found. Most of the residents had 2 or more morbidities at the time of death (52.4 to 74.2%), except in the Netherlands (47.3%), but no significant difference was found between countries.

One month before death, residents in Italy and Poland had the poorest functional and cognitive status (BANS-S mean score of 21.8 and 21.9 respectively; *p* < 0.001) (Table 3).

Clinical complications during the last month of life occurred very often in residents in long-term care facilities in all countries. These mostly consisted of eating or drinking problems (51.9% England, 66.4% Finland and Poland, no significant difference) (Table 3). The proportion of people, according to the nurse, who had pneumonia during the last month of life varied between 21.2% in Poland and 37.7% in the Netherlands (*p* = 0.005). Febrile episodes other than pneumonia occurred in the last month of life most often in Italy (52.4%). Other clinical complications during the last month (hip fracture, stroke, gastrointestinal bleeding and other) varied between 24.2% in Finland and 41.8% in England (*p* = 0.013).

### Differences between types of long-term care facilities within countries

In England, we found no differences in resident characteristics between types of facility. In Italy, having more than 2 morbidities occurred more often in type 2 facilities (84,9%), no other differences were found. Table 4 displays the differences between facility types for the Netherlands and Poland.

**Table 4:** Differences in characteristics of dying facility residents by facility type within countries (*N* = 824)

	NL *N* = 222		PL *N* = 311	
	*N*(%)		*N*(%)	
By facility type^a^	type 1 *N* = 117	type 2 *N* = 94	type 1 *N* = 184	type 2 *N* = 127
Residency before admission to facility^b^				
Own home (living alone or with family or others)	38 (43.2)	57 (73.1)	77 (42.3)	76 (60.8)
General hospital (e.g. acute care hospital)	11 (12.5)	3 (3.8)	93 (51.1)	12 (9.6)
Other facility	20 (22.7)	12 (15.4)	9 (4.9)	26 (20.8)
Other residency (e.g. psychiatric or rehabilitation hospital)	19 (21.6)	6 (7.7)	3 (1.6)	11 (8.8)
*P*-value^c^	**< 0.05**		**< 0.001**	
Length of stay^b^				
Median (min-max) number of days	710 (1–5485)	980 (1–6290)	60 (1–4438)	1007 (3–12,365)
*P*-value^c^	0.742		**< 0.001**	
Type of ward at time of death^b^				
Ward for residents with dementia	64 (59.3)	28 (32.9)	48 (26.2)	8 (6.6)
Ward not specifically for dementia	44 (40.7)	57 (67.1)	135 (73.8)	114 (93.4)
*P*-value^c^	**< 0.05**		0.160	
Place of Death^b^				
Facility	104 (95.4)	67 (80.7)	172 (93.5)	76 (60.3)
Other (e.g. hospital)	5 (4.6)	16 (19.3)	12 (6.5)	50 (39.7)
P-value^c^	0.053		**< 0.001**	
Dementia (yes) ^dfg^	83 (72.2)	43 (45.7)	119 (66.1)	88 (70.4)
*P*-value^c^	**< 0.01**		0.727	
Stage of Dementia (based on CPS/GDS^h^) ^dfk^				
Moderate or mild dementia	17 (21.8)	10 (23.3)	3 (3.6)	14 (21.2)
Severe dementia	21 (26.9)	16 (37.2)	23 (27.4)	14 (21.2)
Very severe or advanced dementia	40 (51.3)	17 (39.5)	58 (69.0)	38 (57.6)
*P*-value^c^	0.415		**< 0.05**	
Diseases at time of death^fi^				
Cancer^f^	11 (13.1)	13 (23.6)	2 (1.2)	8 (9.9)
*P*-value^c^	0.168		**< 0.05**	
Diseases at time of death^fi^				
Cardiovascular disease (not CVA) ^f^	25 (29.8)	19 (34.5)	88 (51.2)	53 (65.4)
*P*-value^c^	0.533		0.160	
Multimorbidities^f^				
0–1 multimorbidities	46 (54.8)	29 (52.7)	75 (43.6)	23 (28.4)
2 multimorbidities	22 (26.2)	15 (27.3)	71 (41.3)	31 (38.3)
3 multimorbidities	13 (15.5)	11 (20.0)	21 (12.2)	19 (23.5)
4 or more multimorbidities	3 (3.6)	0 (0.0)	5 (2.9)	8 (9.9)
*P*-value^c^	0.967		**< 0.05**	
Functional/cognitive status (BANS-S) ^dj^				
Mean (SD)	18.3 (4.7)	16.7 (4.7)	22.9 (3.8)	20.2 (5.1)
*P*-value^c^	**< 0.05**		**< 0.001**	
Global Deterioration Scale Stage 7^dl^	61 (52.1)	37 (40.7)	135 (80.8)	73 (62.4)
*P*-value^c^	0.085		**< 0.01**	

Note: Only variables for which we found a significant difference between facility types in at least one country are reported in this table. Variables for which analyses were conducted but no significant differences between facility types were found are age, gender, diseases at time of death: CVA, pulmonary disease, neurological disease, renal disease, diabetes, other severe disease, CPS and clinical complications: pneumonia, febrile episode, eating or drinking problem and other clinical complication

Percentages may not always add up to 100 because of rounding

Missing values per country: NL: Facility type = 11, ownership = 11 size = 11, admission from = 56, length of stay = 31, type of ward dementia = 29, place of death = 30, dementia = 13, stage of dementia = 18 (83 not applicable because resident did not have dementia), diseases at time of death = 83, multimorbidities = 83

BANS-S = 21 GDS7 = 14

PL: Facility type = 0, ownership = 2 size = 31, admission from = 4, length of stay = 4, type of ward dementia = 6, place of death = 1, dementia = 6, stage of dementia = 63 (98 not applicable because resident did not have dementia), diseases at time of death = 58, multimorbidities = 58

BANS-S = 28 GDS7 = 27

^a^Facility type: In this table only the Netherlands and Poland are reported, since Belgium and Finland only have type 2 facilities and almost no differences between facility types were found in England and Italy. Type 1 includes facilities with 24/7 on-site physicians, nurses and care assistants, type 2 are facilities with 24/7 on-site nurses and care assistants and off-site physicians and type 3 consists of facilities with 24/7 on-site care assistants and off-site nurses and physicians

^b^Reported by administrator/manager of facility

^c^Generalised linear mixed model per country reporting p-value for facility type as a fixed effect, α =0.05. Significant results in bold

^d^Reported by staff member (nurse/care assistant) most involved in care

^f^Reported by treating physician (TP) For 397 out of 1384 residents no questionnaire was returned by the TP, these are not included as missing values below

^g^When either the physician or the nurse (or both) considered the resident to have dementia, this was coded as yes

^h^The variable stage of dementia was based on the scores on the CPS and GDS, as answered by nursing staff, after selecting residents with dementia [16]

^i^Multiple answers possible

^j^Scores on BANS-S range from 7 to 28; higher scores indicate greater severity

^k^Scores on CPS range from 0 to 6; higher scores indicate greater severity

^l^Scores on GDS range from 0 to 6; higher scores indicate greater severity

In the Netherlands, type 1 facilities were larger than type 2 facilities (mean 149 and 95 beds respectively; *p* = 0.031), they had a higher percentage of residents with dementia (72.2 and 45.7% respectively; *p* = 0.004) and poorer functional and cognitive status (*p* = 0.014). In type 2 facilities, residents were more often admitted from their own home (*p =* 0.013) and stayed less often in a dementia ward (*p* = 0.017).

In Poland, type 1 facilities were smaller than type 2 (mean 56 and 104 beds respectively; *p* < 0.001), the length of stay was considerably shorter (median length of stay 60 and 1007 days respectively; *p* < 0.001) due to a difference in admission criteria and the functional and cognitive status of residents was poorer (BANS-S *p* < 0.001). Also, residents of type 1 facilities were more often admitted from a hospital while those in type 2 facilities were more often admitted from their own home (*p* < 0.001) and almost all residents died in the facility (93.5%) while in type 2 facilities residents more often had another place of death (39.7%) such as the hospital (*p* < 0.001).

## Discussion

We found that residents of long-term care facilities currently die at a very old age, on average around 85 years, except in Poland where the mean age of death is 81. Between 80% of the residents in Poland and 90% of the residents in the Netherlands die in the facility. A large proportion of the residents (47–74%) have multiple comorbidities at the end of life and at least 60% have a diagnosis of dementia, often at a very severe or advanced stage. Clinical complications during the last month are frequent and consist mainly of eating or drinking problems (51.9% England; 66.4% Finland and Poland). The average length of stay is relatively short in all countries and varies between 6 months in Poland and Italy and 2 years in Belgium. The highest percentages of residents with poor cognitive and functional status 1 month before death are found in Poland and Italy, where residents also have the shortest length of stay.

This study has several strengths. Firstly, it is the first large-scale study to describe and compare demographic and clinical characteristics of deceased residents of long-term care facilities across 6 European countries. In Italy and Poland this is the first time that nationally representative data have been collected in such facilities. We were able to include 1384 people from 322 facilities in 6 countries, providing cross-country comparable data and giving policy- and decision-makers insight into the key international and national challenges facing the long-term care facility population. Secondly, this study provides an excellent starting point for monitoring changes over time. Thirdly, response rates from all countries were high except in England and non-response analysis shows minimal indication of bias. However, the low numbers of residents included in England contribute to statistical uncertainty for this country. Finally, the use of different proxy respondents allowed for collection of data for many characteristics of the same group of deceased residents.

This study also has some limitations. Firstly, the risk of highly achieving facilities or those with a special interest in palliative care being more prone to participate cannot be excluded. However, due to the proportional stratified random sampling procedure, a nationally (or regionally in the UK and Belgium) representative sample of long-term care facilities in terms of region/province and facility size was achieved. Secondly, the possibility of recall bias cannot be excluded because of the retrospective design of the study. As only deaths from the 3 previous months were included, memory bias is likely to be minimal. Finally, it should be noted that our measure for multimorbidity is based on a predefined list of severe diseases, which explains why the proportion found in our study is lower than in other studies [3].

We found that the large majority of residents of long-term care facilities are female (Table 2). This is not a surprising finding given that females generally live longer [17]. However, this is also likely a feature of them having been widowed and alone for some time, having no one left to care for them at home after their spouse dies. With population ageing and people having to work longer, it is likely that older women will increasingly die in nursing homes, unless greater home support is available.

The length of stay can be considered short in all 6 countries and is shorter now in Belgium and the UK than it was a few years ago [16, 18](there are no trend data available for the other countries). The length of stay is particularly short in Poland and Italy where there is a lower number of beds available in long-term care facilities than in the other countries in our study [19], implying that potential residents have to wait longer to be accommodated. Another reason for later admissions in these countries might be the stronger tradition of informal instead of formal care [20, 21]. The short length of stay found in this study confirms that long-term care facilities are more and more becoming places where people go to live at the very end of their lives when they are highly dependent and have complex health problems. Policy in many countries indeed aims to keep older people at home as long as possible i.e. until they reach very high levels of disability [22]. Given the short length of stay, almost all residents of these facilities can be considered to be at the end of life, making palliative care the most appropriate care approach for this population. Policies supporting these facilities to integrate a palliative care approach may bring substantial benefit to the sector.

It is vital to recognize the complexity and intensity of care that is required, especially in countries like Poland and Italy. Not only is the median length of stay extremely short in these countries (as short as 60 days in type 1 facilities in Poland), making the delivery of high quality palliative and end-of-life care more challenging [23], but we also found the highest levels of cognitive and functional impairment as well as very high levels of advanced dementia among the residents at the time of death. At the same time, another study based on data from the PACE project shows that palliative care knowledge among Polish and Italian nurses and care assistants working in long-term care facilities is deficient [24]. There is also evidence that the integration of palliative care in long-term care facilities in Poland and Italy is minimal if not non-existent, especially compared with countries like the UK, the Netherlands and Belgium [7]. National policies focusing on enhancing palliative care development in long-term care facilities are particularly needed in Poland and Italy to ensure optimal levels of care.

The results of our study are in line with recent findings in the literature on the rise of chronic diseases [25] and increasingly complex care needs among residents of long-term care facilities [26, 27]. We found that a large proportion of residents have multiple morbidities, many have considerable cognitive and functional impairment in the last month of life (reflected in high levels of dependency as shown by mean BANS-S scores ranging from 17.5 in the UK to 21.9 in Poland), and at least 60% die with dementia (Table 3). This has huge implications for the care that they need. Caring for residents at the end of life entails a high burden of care for the nurses and care assistants working in this setting, and places extremely high demands on their knowledge, confidence and skills in providing palliative care [28, 29]. The quality of care is thus highly contingent on staff and is a major concern of long-term care facilities [30]. Giving rising costs and demand, understanding how to meet the increasingly complex needs of residents efficiently, and determining and providing the appropriate numbers and type of staff (skill mix) and the education and training in palliative care that they need should be a high public health priority. Inadequate skill mix in staff has been linked to low-quality care [30]. Research therefore needs to investigate which staff skills contribute to high-quality palliative care. This study highlighted various complex health problems of nursing home residents (dementia, comorbidities, eating and drinking problems and poor cognitive and functional status). Education of nursing staff should focus on learning skills to handle these health problems. Finally, given that so many residents suffer from advanced dementia at the time of death, we also recommend that policies addressing this sector highlight the need for the integration of high-quality palliative care together with high-quality dementia care, enabling long-term care facilities to become centers of excellence in dementia end-of-life care.

## Conclusion

Although there are important country differences, the population currently living and dying in long-term care facilities is very complex, displaying multiple diseases with considerable cognitive and functional impairment and high levels of dementia. Given the complex care needs of long-term care facility residents, palliative care is the most appropriate care approach for this population and education of nursing staff should include learning skills to meet these needs. Since many residents also suffer from advanced dementia at the time of death, we recommend that policies addressing this sector highlight the need for the integration of high-quality palliative care together with high-quality dementia care. This study is an excellent starting point for monitoring populations of people who die in long-term care facilities. The current challenges of dying in such facilities need adequate policy and practice responses as soon as possible.

## Data Availability

The datasets used and/or analysed during the current study are available from the corresponding author on reasonable request.

## Abbreviations

BANS-S: The Bedford Alzheimer Nursing Severity Scale
CPS: The Cognitive Performance Scale
GDS: The Global Deterioration Scale
PACE: Palliative Care for Older People in Europe
